# External fixation of femoral defects in athymic rats: Applications for human stem cell implantation and bone regeneration

**DOI:** 10.1177/2041731413486368

**Published:** 2013-04-12

**Authors:** Terasa Foo, Jeffrey Reagan, John T Watson, Berton R Moed, Zijun Zhang

**Affiliations:** 1 Department of Orthopaedic Surgery, Saint Louis University, St. Louis, MO, USA; 2 Orthobiologic Laboratory, Union Memorial Hospital, Baltimore, MD, USA

**Keywords:** External fixator, bone defect, athymic rat, bone regeneration, stem cells

## Abstract

An appropriate animal model is critical for the research of stem/progenitor cell therapy and tissue engineering for bone regeneration in vivo. This study reports the design of an external fixator and its application to critical-sized femoral defects in athymic rats. The external fixator consists of clamps and screws that are readily available from hardware stores as well as Kirschner wires. A total of 35 rats underwent application of the external fixator with creation of a 6-mm bone defect in one femur of each animal. This model had been used in several separate studies, including implantation of collagen gel, umbilical cord blood mesenchymal stem cells, endothelial progenitor cells, or bone morphogenetic protein-2. One rat developed fracture at the proximal pin site and two rats developed deep tissue infection. Pin loosening was found in nine rats, but it only led to the failure of external fixation in two animals. In 8 to 10 weeks, various degrees of bone growth in the femoral defects were observed in different study groups, from full repair of the bone defect with bone morphogenetic protein-2 implantation to fibrous nonunion with collagen gel implantation. The external fixator used in these studies provided sufficient mechanical stability to the bone defects and had a comparable complication rate in athymic rats as in immunocompetent rats. The external fixator does not interfere with the natural environment of a bone defect. This model is particularly valuable for investigation of osteogenesis of human stem/progenitor cells in vivo.

## Introduction

Fracture nonunions and bone defects are difficult to treat. Stem/progenitor cells and tissue engineering have demonstrated great potential for enhancing bone regeneration and fracture healing. The promising osteogenic stem/progenitor cells and products of bone tissue engineering require validations in vivo as the complexity of bone-forming environment cannot be duplicated in vitro at the present time. Animal study, therefore, is a necessity to translate the stem/progenitor cell therapy and tissue engineering technology to potential human clinical applications. Over the years, animal models of fractures and bone defects have been developed and utilized in a variety of experimental studies.^1^ In these models, the methods of bony fixation contribute greatly to the environment of bone formation and influence the outcome of the studies.^2^

Human stem/progenitor cells are highly relevant to clinical applications. However, transplantations of human stem/progenitor cells into fracture sites and bone defects in animals face rejection by the host due to the major immune barrier between species. Immunodeficient animals can better tolerate xenografts, but they are generally believed susceptible to infection.^3,4^ We have designed an external fixator to stabilize bony defects for the purpose of implantation of human stem/progenitor cells and applied it to critical-sized femoral defects in a total of 35 athymic rats. In this article, we provide details of assembly and application of this external fixator and the results of using this external fixator in several separate experiments involving implantation of human stem/progenitor cells. The effectiveness of each treatment in bone regeneration, however, is not a primary focus of this article. The applicability of this device in athymic rats was investigated by assessing the stability of the bony defect, bone healing and infection rates.

## Materials and methods

### Design of external fixator

**Figure 1.**
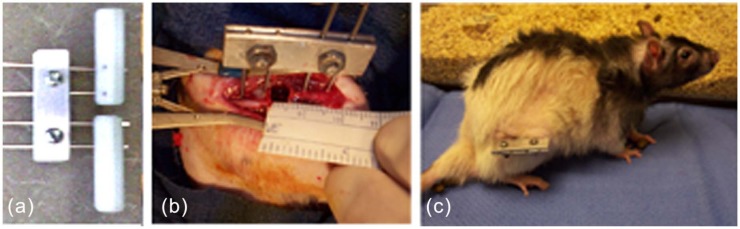
(a) Prototype of the external fixator. Plastic dowels represent bone-ends. (b) During surgery, the external fixator was assembled and a bone defect was created between the two inner pins. (c) A rat applied with an external fixator for a femoral defect and standing on the operated limb.

A 4-pin monolateral external fixator was developed (Figure 1(a)) using threaded Kirschner wires (diameter 1.1 mm, Synthes, West Chester, PA, USA) as pins. The external fixation pin clamp was made with readily available 1/8-in aluminum stock from a hardware store. Two 8 mm × 35 mm aluminum rectangles were fashioned to form a “sandwich”-style clamp. Four pin grooves measuring 1 mm wide × 0.5 mm deep were machined into one side of each pair of the aluminum clamps to prevent pins from migrating laterally in the clamp. The distance between the two medial pins was 10 mm allowing spanning of the defect, and the distance between the proximal and distal pair of pins was 7 mm to provide adequate fixation on each side of the defect. The clamp was fitted with two M4-size fasteners to secure the clamp over the wires. A typical assembled external fixator weighed 4.0 g.

### Surgery

All animal experiments were approved by the Saint Louis University Institutional Animal Care and Use Committee. A total of 35 male athymic nude rats (300–350 g, National Cancer Institute, Frederick, MD, USA) underwent a surgery to apply the external fixator and create a critical-sized defect. Ketamine (70 mg/kg) and Xylazine (9.0 mg/kg) were used for intraperitoneal anesthesia. Animals received Baytril (enrofloxacin) at a dose of 5 mg/kg preoperatively and once daily postoperatively for 2 days, as well as buprenophine hydrochloride (0.1 mg/kg) for pain relief pre- and postoperatively. Using aseptic technique, a lateral approach was used to expose the right femur, from the greater trochanter to the lateral femoral condyle, through the interval between the vastus lateralis and biceps femoris. The previously described 4-pin linear external fixator was then applied. Threaded tip 1.1-mm Kirschner wires were inserted into the femur with a power drill, using the clamp of external fixator as a guide. After the external fixator was assembled, a 6-mm critical-sized defect was created using a burr between the two medial pins in the mid-shaft of the femur under continuous saline irrigation (Figure 1(b)). Periosteum at the bone-ends was kept intact. The wound was then copiously irrigated with normal saline and explored to ensure removal of bony materials. Human mesenchymal stem cells or human endothelial progenitor cells (1 × 10^6^) were suspended in collagen solution (Millipore, Billerica, MA, USA) and incubated at 37°C for 1 h for gelation. Collagen gel (200 µL) with or without cells was transferred to the bone defect with a pipettor. Recombinant human bone morphogenetic protein-2 in collagen sponge (BMP-2, Infuse^®^, Medtronic, Fridley, MN, USA) was trimmed into the shape of bone defect and implanted. The fascial layer was closed in an interrupted fashion with 5.0 Monocryl^™^ suture (Ethicon^™^) to keep the collagen gel in place in the bone defect. Rats were then returned to individual cages without immobilization (Figure 1(c)). The animals received antibiotics and pain medication as previously described. Postoperative radiographs were taken weekly to assess bone formation as well as the stability of the external fixator. At 8-10 weeks, the rats were euthanized. The affected femur of each rat was then dissected and fixed with 4% paraformaldehyde. After decalcification in 10% ethylenediaminetetraacetic acid (EDTA), the femoral tissue blocks were sectioned using a cryostat. Tissue sections were stained with hematoxylin and eosin and examined under a light microscope for evidence of bone formation and tissue reactions.

## Results and discussion

**Figure 2.**
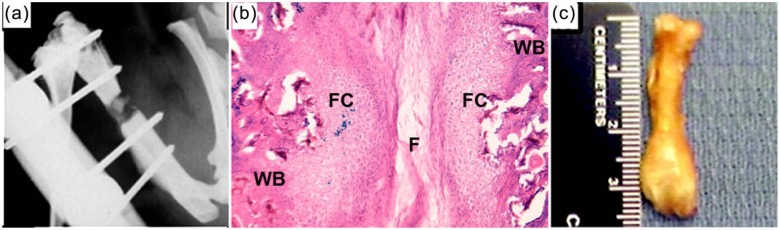
(a) Radiograph of a rat that was implanted with BMP-2 in the femoral defect shows callus within the segmental defect at week 1. (b) Histological section of hematoxylin and eosin (H & E) staining shows endochondral ossification in the femoral defect at week 8 after implantation of human endothelial progenitor cells (F: fibrous tissue; FC: fibrocartilage; WB: woven bone). (c) An example of rat femur. The femoral defect is completely repaired at week 10, after BMP-2 implantation and external fixation.

To examine the osteogenic capacity of stem/progenitor cells in bone defects, two local factors are critical: the length of the bony defect and the mechanical stability of the bone. Since bone is capable of self-regeneration to a certain length, numerous studies have explored the concept of critical size of defects (CSD) at different anatomical locations and in various animal species with the goal of preventing concomitant bone healing.^1^ For rats, a 6-mm defect in femur has been suggested as an appropriate CSD.^5,6^ In the current animal model, when only collagen gel was implanted, slight callus formation was observed at each bone-end of the defects in week 3, and no further propagation of healing was seen in the following weeks, a sign of atrophic nonunion. The defects were unchanged at week 8 in all 8 animals that were implanted with collagen gel alone. Therefore, the femoral CSD for this particular species of immunodeficient rats fixed with an external fixator is 6 mm. Rats that received BMP-2 implantation at the defect site had full healing of the defect, confirming the applicability of this model for investigating bone regeneration (Figure 2(c)).

After CSD is created, fixation to sustain the length of defect and stabilize bone-end is required. Various methods have been developed using common orthopedic techniques to fix bone defects in animals, including intramedullary Kirschner wire fixation, internal (plate) fixation,^7,8^ and external fixation.^1^ Intramedullary Kirschner wire fixation lacks a locking mechanism to prevent interfragmentary movement, which causes the reduction of the length of the defect.^9,10^ Internal plate fixation, if applied appropriately, can provide adequate mechanical stability to the bone defect.^5^ However, if the hardware is not specially manufactured for rat femurs and not applied with specialized tools, the fixation may not be able to achieve the desired stability. In addition, the skills and scientific principles of internal fixation require vigorous training. Importantly, intramedullary pins and plates are foreign materials within the bony defect, which interferes with the local physiological environment.^11^ Alterations of tissue environment are particular concerns for stem/progenitor cells, which are multipotent and differentiated according to environmental cues.^12^ The use of external fixation avoids the contact between fixation materials and local tissues within the defects, thus sparing the implanted stem/progenitor cells from potential exposure to nonphysiological stimulations. The application of an external fixator is also relatively easy and does not require specialized training.

To our knowledge, there are no commercial products available for external fixation of rat bony defects. This has led many investigators to develop their own systems of external fixation.^7,10,13^ For practical reasons, the external fixator must be lightweight, inexpensive, relatively easy to apply, and stable through the duration of the experiment. An assortment of external fixation frames and techniques has been developed for this purpose. While bone cement is convenient to apply and provides initial solid fixation to the pins, it has been found that there is often loosening in late stage of the fixation. The external fixator used in this study was designed for convenient assembly without the use of highly specialized tools or skills. The parts for this external fixator can be purchased from any hardware stores. The external fixator provided sufficient strength of bone fixation using a lightweight material (aluminum alloy) that the animals were able to tolerate without difficulty. The ability to bear weight early is important for bone regeneration in long bone defects as it provides mechanical stimulation.^14^ In this study, all animals had the ability to bear weight immediately postoperatively and walked without difficulty. The external fixator used in the current study is also adjustable, and the M4 fasteners can be tightened as needed to prevent loosening of the frame.

The most common complications of external fixation are pin loosening and pin tract infection.^15^ Pin tract infection was observed in nine rats and all healed after local daily application of antibiotic ointment. Two rats developed deep tissue infection, one of which necessitated euthanasia. Pin loosening was observed in nine rats. Most of the cases were corrected by adjustment of the external fixator and did not affect the overall stability of the device. Only two of the external fixators failed, both of these failures occurred at 7 weeks post operation. One rat died 4 weeks post operation of unknown causes; however, it did have deep infection found on necropsy. One rat developed a femur fracture at the proximal pin site at 3 weeks, which was likely iatrogenic during the insertion of pins.

It is evident that this self-designed external fixator provided a stable mechanical environment for bone regeneration in the defects. The fact that BMP-2 implantation had healing of the defect proves that there was adequate mechanical stability of the defect to allow for potential healing. Defects implanted with stem/progenitor cells showed various degrees of bone regeneration at the defect site (Figure 2(a)). On histologic analysis, regenerated woven bone and fibrocartilage were seen within the defects implanted with human stem/progenitor cells (Figure 2(b)). This indicates new bone was regenerated through the process of endochondral ossification. In the tissues surrounding the defect, there were no obvious signs of inflammatory reaction.

When an external fixator is applied to immunodeficient animals, such as the athymic rats used in this study, infection is a concern. Among the 35 athymic rats that had an external fixator applied to stabilize the critical-sized femoral defects, only two rats had deep infection. None of the rats with superficial pin tract infection developed uncontrollable systematic infection or required additional antibiotic treatment beyond topical antibiotic ointment. The rate of the failure of external fixation in this study was consistent with the 5% rate of failure for applying external fixators to the bone defects (without cell implantation) in immunocompetent laboratory rats.^16^ Being able to tighten the external fixator during the course of follow-up is one of the advantages of this type of external fixator and attributes to the low failure rate of external fixation, as compared to a high failure rate (8/8) of internal fixation in 8 weeks.^7^

In summary, an external fixator has been developed and successfully applied to critical-sized femoral defects in athymic rats. This model is particularly useful for studying bone regeneration with human stem/progenitor cells in vivo.
